# Oxygen as a catalyst in the Earth's interior?

**DOI:** 10.1093/nsr/nwab009

**Published:** 2021-01-16

**Authors:** Yanhao Lin, Wim van Westrenen

**Affiliations:** Center for High Pressure Science and Technology Advanced Research, China; Earth and Planets Laboratory, Carnegie Institution for Science, USA; Department of Earth Sciences, Faculty of Science, Vrije Universiteit Amsterdam, the Netherlands

The abundance of the element oxygen is not only crucial for life at the surface of the Earth, but also greatly affects the properties and dynamics of the interior of our planet. The combination of a highly oxidized atmosphere and a large reduced metallic core makes our Earth unique among the terrestrial planets, and has led to substantial variations and gradients in oxygen abundances in the rocky crust and mantle. Recent results from a range of research fields including mineral physics, seismology, thermodynamic modelling and numerical geodynamic simulations have provided compelling evidence that the oxygen distribution in Earth's mantle is highly heterogeneous, with large oxygen-rich domains within a generally reducing mantle environment [1–3], with possible variations up to over 10 orders of magnitude expressed through oxygen fugacity. A novel deep super-oxidized form of FeO_2_, produced by pressure-induced decomposition of goethite (FeO_2_H) and releasing hydrogen, was recently revealed based on theoretical and experimental studies at the high pressure-temperature conditions present in Earth's lower-mantle [1]. In addition, an oxygen-rich layer can be produced and thickened when water transported into the mantle by subducting slabs is brought into contact with the nearly inexhaustible metallic iron source in the core [2,4].

A key question coming out of these recent results is: can oxygen, as the most abundant element in the Earth and essential structural component in the silicate and oxide minerals characterizing the terrestrial interior, be the catalyst linking the deep Earth with large-scale, geologically rapid events on the Earth's surface (e.g. Wilson cycles, the atmosphere's great oxidation, the formation of large igneous provinces, and snowball Earth events)? For example, sustained accumulation of metastable oxygen-rich patches over long periods of geological time early in Earth's history would eventually lead to a tipping point, at which an outburst of oxygen-rich material could occur at the core–mantle boundary. Transporting such oxygen-rich material to the surface could, through a range of chemical reactions, contribute to the oxygenation of Earth's atmosphere [3].

**Figure 1.**
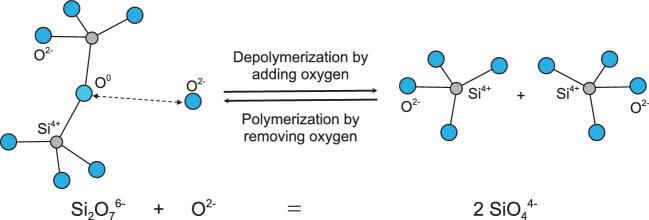
Schematic reaction mechanism of (de)polymerization of silicate magma by adding/removing oxygen.

To quantify the role of oxygen in linking the deep Earth with the evolution of Earth's surface and atmosphere, the effect of oxygen on melting of rocks needs to be assessed. To date, studies of chemical compositional effects on melting of mantle rocks has focused mostly on changes in the oxidation state of iron atoms, and on the concept of melting temperature decreases due to the addition of volatile compounds (e.g. H_2_O, CO_2_, CH_4_, H_2_) to Earth's mantle, e.g. [5–8]. Volatile-induced melting has been generally accepted as an important process for a long time, especially in subduction zone settings, which provide a straightforward process to inject volatile compounds into the source region of subduction zone volcanoes, but the role of the oxygen atoms in H_2_O and CO_2_ has not been assessed in detail. Considering that the major reactions determining the solubility of hydrogen and carbon in silicate melts involve the breaking and/or formation of bonds with oxygen, even in the case of reduced compounds such as CH_4_ or H_2_, e.g. [7,8], the role of oxygen itself warrants more attention. We propose that the abundance of oxygen can play a key role in changing the solidus and liquidus temperature of silicate rocks. This hypothesis can be depicted graphically (Fig. 1) by assessing the role O^2−^ could play in the depolymerization of silicate chains in silicate melts.

We note that the reaction depicted in Fig. 1 does not depend on the valence state of iron in magma, nor does it depend directly on the presence of volatile carbon- or hydrogen-bearing compounds. In our view, the valence state variations of iron (that are commonly studied when effects of oxygen fugacity on mantle rocks are assessed) could be a manifestation of oxygen abundance (i.e. oxygen fugacity) variations, not a main reason in itself for variations in melting behaviour of mantle rocks. According to our hypothesis, oxygen has the potential to be a chemical driving force, depolymerizing silicate magma and hence lowering the solidus and liquidus of mantle rocks. An effect of oxygen fugacity on mantle melting could link perturbations in steady-state thermally driven mantle convection, leading to super plumes and catastrophic formation and rifting of supercontinents, to variations in oxygen fugacity in Earth's mantle. If our hypothesis is proven correct, it has far-reaching consequences for melting in other rocky bodies too, including Mars (which has a more oxygen-rich interior than the present-day bulk silicate Earth) and Mercury and the Moon (which have oxygen-depleted interiors compared to the present-day silicate Earth). This new concept could help understand the grand picture of the evolution of the entire Earth and the link between the interior and the atmosphere.
